# MicroRNA-195 regulates proliferation, migration, angiogenesis and autophagy of endothelial progenitor cells by targeting GABARAPL1

**DOI:** 10.1042/BSR20160139

**Published:** 2016-10-14

**Authors:** Jianwen Mo, Daifen Zhang, Renze Yang

**Affiliations:** * Department of Orthopedics Surgery, the First Affiliated Hospital of Gannan Medical University, Ganzhou 341000, Jiangxi Province, P.R. China

**Keywords:** autophagy, endothelial progenitor cells, GABARAPL1, *miR-195*

## Abstract

Deep vein thrombosis (DVT) is a common type of venous thrombosis. Successful resolution of DVT-related thrombi is important in the treatment of DVT. Endothelial progenitor cells (EPCs) have emerged as a promising therapeutic choice for DVT-related thrombus resolution; however, the clinical application of EPCs faces many challenges. In the present study, the expression of *miR-582*, *miR-195* and *miR-532* under hypoxic or normoxic conditions was measured using quantitative real-time PCR analysis (qRT-PCR) and the results showed that the increased fold of *miR-195* was highest in human EPCs (hEPCs) under hypoxic conditions. Then the role and regulating mechanism of *miR-195* in improving the function of EPCs was investigated. To investigate the effect of *miR-195* inhibition on the autophagy of hEPCs, the expression of the autophagy-related genes LC3B and beclin1 was examined using western blotting, and the formation of autophagosomes was observed using TEM. The results indicated that the inhibition of *miR-195* expression could promote autophagy of hEPCs. In addition, we investigated the role of *miR-195* on the proliferation, migration and angiogenesis of hEPCs under hypoxia. The results revealed that *miR-195* inhibition promotes cell proliferation, migration and angiogenesis of hEPCs under hypoxia. Furthermore, GABA type A receptor associated protein like 1 (GABARAPL1) was identified as a directed target of *miR-195* and GABARAPL1 silencing could decrease the effect of *miR-195* knockdown on cell proliferation, migration, angiogenesis and autophagy of hEPCs under hypoxia. Together, these results indicate that *miR-195* regulates cell proliferation, migration, angiogenesis and autophagy of hEPCs by targeting GABARAPL1.

## INTRODUCTION

Blood clots that form within a vein are referred to as venous thrombi. The formation of a blot clot in the deep leg veins is termed deep vein thrombosis (DVT). DVT could lead to pulmonary hypertension, recurrent thrombosis or even fetal pulmonary embolism [1], and is a common complication of surgery. Although many treatment options for DVT including anticoagulation, pharmacologic thrombolysis, endovascular and surgical interventions and physical measures have shown promise, they also have a number of side effects, such as the risk of bleeding and wound complications [2,3]. Successful resolution of DVT-related thrombi is important in the treatment of DVT. As such, the development of a promising novel therapy for DVT-related thrombus resolution is essential.

A special type of stem cells, endothelial progenitor cells (EPCs), which have capacity to proliferate, migrate and form new vessels through differentiation into endothelial cells [4–6], have recently, emerged as a promising therapeutic choice for DVT-related thrombus resolution. When DVT occurs, EPCs could hone in on and incorporate themselves into the site of the injured vessel and thrombi [7]. The mobilization of EPCs can remarkably increase new blood vessel formation [1] and improve thrombus resolution [8]. Although EPCs show good therapeutic potential, their clinical application faces many challenges. Therefore, investigation of a method to improve the function of EPCs is being widely discussed.

miRNAs are a novel class of 21–24 nucleotide long, endogenous, non-coding small ribonucleic acids that function in RNA silencing and post-transcriptional regulation of gene expression by binding to the 3′UTR of target genes [9,10]. Previous studies have demonstrated that miRNAs, including *miR-150*, *miR-126*, *miR-145*, play an important role in regulating the function of EPCs [11–13]. In another previous study, a panel of miRNAs, such as *miR-582*, *miR-195* and *miR-532*, were identified as new biomarkers for the detection of DVT [14]. However, the expression and function of *miR-582*, *miR-195* and *miR-532* in EPCs, especially in hypoxic conditions such as that of DVT, remains unknown. In the present study, we found that, the increased fold of *miR-195* was highest in human EPCs (hEPCs) under hypoxic conditions among these three miRNAs. And the inhibition of *miR-195* expression could promote autophagy of EPCs. Furthermore, we investigated the role of *miR-195* on the proliferation, migration and angiogenesis of EPCs under hypoxia, and identified GABA type A receptor associated protein like 1 (GABARAPL1) as a directed target of *miR-195*.

## MATERIALS AND METHODS

### Isolation and culture of hEPCs

Blood samples were collected from two female and three male healthy volunteers (average age, 27±5.2). All study protocols were approved by the Research Ethics Board of the First Affiliated Hospital of Gannan Medical University. All healthy volunteers gave informed consent to participate in the study. Isolation and culture of hEPCs were carried out as described previously [15]. Briefly, a 20 ml sample of venous blood was used for the isolation of hEPCs. Samples were processed within 4 h of collection, and peripheral-blood mononuclear cells were isolated by Ficoll density-gradient centrifugation. Recovered cells were washed twice in PBS, and centrifuged. The cell pellet was suspended in endothelial basal growth medium supplemented with EGM-2 MV SingleQuots and 5% heat inactivated FBS (Lonza). The solution was then plated in a 24-well plate coated with 10 μg/ml human plasma fibronectin (FN, Millipore). After removing unbound cells at 96 h, the bound cell fraction was maintained in above culture medium. Spindle-shaped cells were observed after 7 days. Colonies of endothelial-like cells were grown to confluence, and subsequently trypsinized and plated uniformly on to a new 24-well plate as the first passage. The medium was changed every 3 days. At 80% confluence, cells were harvested with 0.25% trypsin and passaged at a ratio of 1:2. Subsequent passages were performed similarly. Passages 3–6 hEPCs were used in the present study.

### Synthesis of miRNA mimics, GABARAPL1 siRNA, transfection and hypoxic exposure

The *miR-195* mimic, *miR-195* inhibitor mimics and negative control (NC) mimics were purchased from Ribobio Co. A small interfering RNA against GABARAPL1 (si-GABARAPL1) and NC (si-NC) were purchased from Santa Cruz Biotechnology. Cells were plated at 50% confluence and transfected with 100 nM mimic or siRNA using Lipofectamine® RNAiMAX Transfection Reagent (Invitrogen) according to the manufacturer's protocol. Cells were harvested 24 or 48 h after transfection for further analysis. For exposure of cells to hypoxia, cells were cultured in a Billups-Rothenburg chamber with 94% N_2_, 1% O_2_ and 5% CO_2_ at 37°C for a certain period.

### RNA extraction and quantitative real-time PCR analysis

Total RNA was extracted from harvested cells using TRIzol reagent (Invitrogen). To analyse *miR-582*, *miR-195* and *miR-532* expression, reverse transcription PCR was performed using specific stem-loop reverse transcription primers, miRNA first strand synthesis was performed using a First Strand Synthesis Kit (Takara), and quantitative real-time PCR analysis (qRT-PCR) was performed using a SYBR Green Real time PCR Master Mix (Toyobo) on an Applied Biosystems 7500 system (Applied Biosystems). U6 was used as an internal control. To quantify mRNA levels of GABARAPL1, reverse transcription PCR was performed using the PrimeScript RT Reagent Kit with cDNA Eraser (Takara), and qRT-PCR was performed using a SYBR Green Realtime PCR Master Mix (Toyobo). 18s rRNA was used as an internal control. The primer sequences used in qRT-PCR are shown in Table 1. Gene expression was measured in triplicate, quantified using the 2^−ΔΔCT^ method and normalized to a control.

**Table 1:** Primers used for qRT-PCR F: forward primer, R: reverse primer.

Primer name	Sequence (5′–3′)
*miR-195-F*	ACACTCCAGCTGGGAGCTACATCTGGCTACTG
*miR-195-R*	TAGCAGCACAGAAATATTGGC
*miR-582-F*	ACACTCCAGCTGGGTTACAGTTGTTCAACCAGT
*miR-582-R*	CTCAACTGGTGTCGTGGA
*miR-532-F*	ACACTCCAGCTGGGCCTCCCACACCCAAGGCT
*miR-532-R*	CTCAACTGGTGTCGTGGA
U6-F	CTCGCTTCGGCAGCACA
U6-R	AACGCTTCACGAATTTGCGT
GABARAPL1-F	ATTGTAGAGAAGGCTCCAAA
GABARAPL1-R	CCTCATGATTGTCCTCATAC
18s rRNA-F	CCTGGATACCGCAGCTAGGA
18s rRNA-R	GCGGCGCAATACGAATGCCCC

### Transwell migration assay

The migration of hEPCs was evaluated using a transwell migration assay. Briefly, 1×10^5^ cells were suspended in 200 μl of EBM-2 medium without EGM-2 SingleQuots and placed in the upper chamber of an 8.0 μm pore size transwell plate (BD Biosciences). After incubation for 24 h in a Billups-Rothenburg chamber with 94% N_2_, 1% O_2_ and 5% CO_2_ at 37°C, the cells that failed to migrate were removed from the upper surface of the filters using cotton swabs, and cells that migrated to the lower surface of the filters were stained with 0.1% crystal violet stain solution. Migration was determined by counting the cell number with a microscope (Olympus). The average number of migrating cells in five fields was taken as the cell migration number of the group. All experiments were repeated three times.

### Matrigel tube formation assay

Growth factor-reduced Matrigel (BD Biosciences) was added into 96-well plates (50 μl/well) and allowed to solidify in order to analyse the capillary-like tube formation of hEPCs. Passage 3 hEPCs were seeded into 24-well plates. hEPCs (1×10^4^) were resuspended in 200 μl of EBM-2 without EGM-2 SingleQuots supplement and then plated on Matrigel. After 18 h, tube images were obtained using an inverted microscope (Leica). The degree of tube formation was quantified by measuring the number of tubes in three random fields from each well. All experiments were repeated three times.

### Cell proliferation assay

Cell proliferation was monitored using the CellTiter 96® AQueous One Solution Cell Proliferation Assay kit (Promega), performed according to the manufacturer's protocol. Briefly, 24 h after transfection, hEPCs were seeded at 1×10^4^ per well in 96-well plates. The cell proliferation assay was performed on days 1, 2 and 3. Twenty micolitres of CellTiter 96® AQueous One Solution Reagent (Promega) was added to each well, and the plate was incubated for 4 h at 37°C. Prior to the endpoint of incubation, the absorbance was measured at 490 nm using a microplate reader (multiscan MK3; Thermo Fisher Scientific). Each sample was assayed in triplicate. The proliferation ratio was calculated using the following formula: proliferation rate=survival rate=(*D*_test_/*D*_negative control_) × 100%. All experiments were performed in triplicate and repeated three times.

### TEM

At the end of the intervention, hEPCs from each group were digested with 0.25% trypsin and collected in centrifuge tubes, followed by centrifugation at 400 ***g*** for 10 min and then by a second centrifugation at a speed of 300 ***g*** for an additional 10 min. The supernatant was discarded, and 2.5% glutaraldehyde was added to the tubes to fix the cells. After 2 h of fixation, dehydration, embedding, sectioning and staining were performed using the normal methods. Autophagosomes were observed under a TEM (JEM-1010, Matsunaga Manufacturing) and photographed. All experiments were repeated three times.

### Western blotting

Each group of hEPCs was lysed using RIPA buffer (Beyotime Biotechnology). The total protein concentration was determined using a BCA Protein Assay kit (Beyotime Biotechnology). Thirty micrograms of protein was loaded and separated on 10% SDS/PAGE, and transferred to PVDF membranes (Millipore). Membranes were blocked for 1 h at room temperature with 5% milk in TBS containing 0.05% Tween-20 (TBST), incubated for 1 h with rabbit polyclonal anti-beclin 1 (1:500, ab55878), rabbit polyclonal anti-LC3B (1:800, ab48394), rabbit polyclonal anti-GABARAPL1 (1:800, ab86497) or rabbit polyclonal anti-GAPDH (1:2500, ab9485) primary antibodies (all antibodies were purchased from Abcam), and washed three times with TBST. Membranes were incubated with horseradish peroxidase-conjugated goat anti-rabbit IgG H&L secondary antibody (1:10000, ab97080, Abcam) for 40 min, washed three times with TBST and proteins were visualized using ECL (Thermo Scientific Pierce ECL Plus). The film was scanned and the densitometric analysis was performed using QuantityOne software (Bio-Rad Technologies). For the quantification of specific bands, the same size square was drawn around each band to measure the density and then the value was adjusted by the density of the background near that band. The results of densitometric analysis were expressed as a relative ratio of the target protein to reference protein. All experiments were repeated three times.

### Reporter vector construction and luciferase reporter assay

The miRNA target predication software Targetscan (http://www.targetscan.org) and miRanda (http://www.microrna.org/microrna/home.do) were used to predict the targets of *miR-195*. The full-length wild-type 3′UTR of GABARAPL1 (NM_031412 was amplified using primers XhoI-Fand NotI-R, and cloned into the psi-CHECK-2 vector (Promega). The mutant-type 3′UTR of GABARAPL1 was obtained through PCR-based site-directed mutagenesis using primers mut-F and mut-R. The primer sequences used in the reporter vector construction are shown in Table 2. All inserts and plasmids were verified by DNA sequencing. 293T cells, plated on 24-well plates, were co-transfected with 100 ng plasmid and 200 nM *miR-195* mimic or miR-NC. Cell lysates were harvested 48 h after transfection, and firefly and *Renilla* luciferase activities were measured by the Dual-Luciferase Reporter Assay System (Promega), according to the manufacturer's instructions. Three independent experiments were performed.

**Table 2:** Primers used for luciferase reporter construction F: forward primer, R: reverse primer.

Primer name	Sequence (5′–3′)
XhoI-F	ccgctcgagGTGGTTGGAAGCCCAGCAGATGGGAG
NotI-R	ataagaatgcggccgcTAAGGTAAGCAATTATTTTATTTC
mut-F	TTTCACATGCTCAATTGATATTTTTACGACGATCCTCGGCCCAGGGAGAAAGCATGT
mut-R	ACATGCTTTCTCCCTGGGCCGAGGATCGTCGTAAAAATATCAATTGAGCATGTGAAA

### Statistical analysis

All statistical analyses were performed using SPSS 19.0 software (IBM). Results are represented as means ± S.D. A Student's *t* test was used to compare means from different groups; *P* values <0.05 were regarded as statistically significant.

## RESULTS

### Expression of *miR-195* is increased under hypoxic conditions in hEPCs

**Figure 1:**
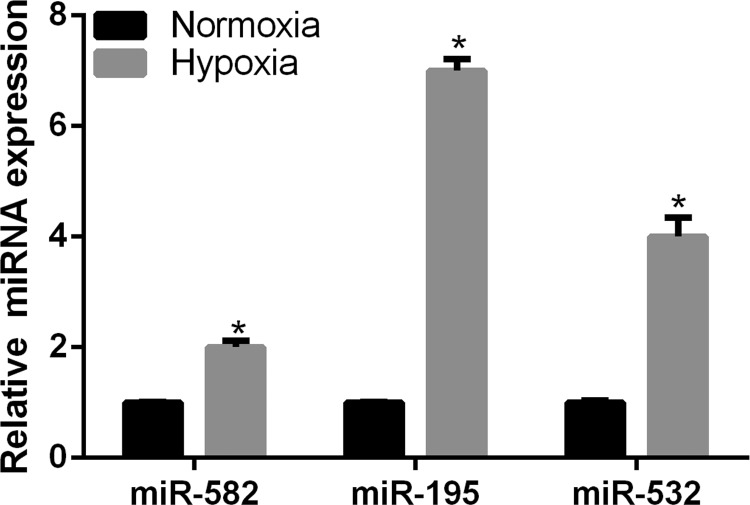
The expression of *miR-582*, *miR-195* and *miR-532* under normoxic and hypoxic conditions as detected by qRT-PCR (*P<0.05)

hEPCs were cultured under normoxic and hypoxic conditions for 24 h. The hEPCs were subsequently harvested for qRT-PCR analysis. The results of qRT-PCR showed that the expression of *miR-582*, *miR-195* and *miR-532* was all significantly increased under conditions of hypoxia compared with that under normoxia (Figure 1). Among these three miRNA, only *miR-195* was demonstrated to have functions in regulating cell migration, angiogenesis and autophagy in many kinds of cells [16–20]. In addition, only *miR-195* was reported to have function under hypoxia condition [21]. Furthermore, the increased fold of *miR-195* was highest under hypoxic conditions in hEPCs. Based on above reasons, *miR-195* was chose for the following study.

### Knockdown of *miR-195* promotes the autophagy of hEPCs under hypoxia

**Figure 2:**
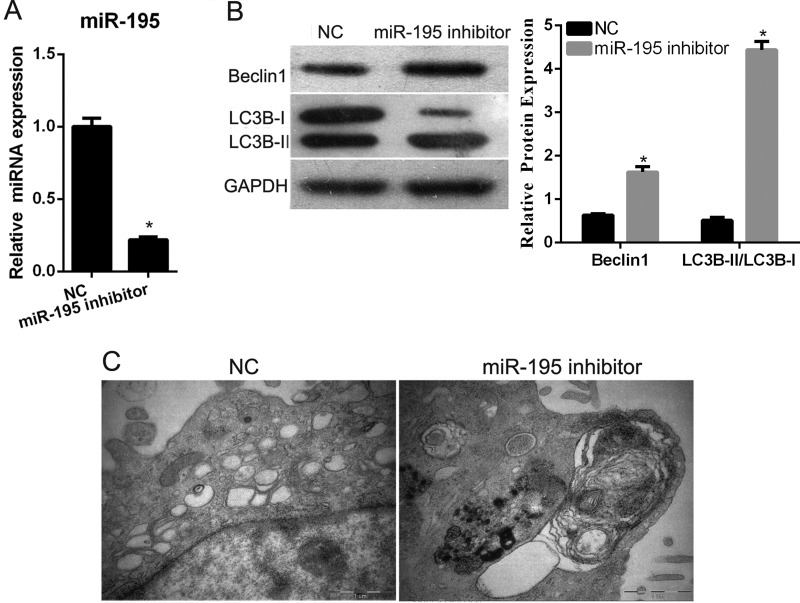
Knockdown of *miR-195* promotes the autophagy of hEPCs under hypoxia (**A**) The expression level of *miR-195* after *miR-195* inhibitor or NC transfection as detected by qRT-PCR. (**B**) Protein expression level of autophagy-related gene LC3B and beclin1 as examined by western blotting. Left is representative images, and right is quantification of conversion of LC3B-I to LC3B-II (LC3B-II/LC3B-I) and beclin1 expression in each group presented in bar graphs as fold-increase. (**C**) Representative TEM images from each group, wherein the cells were cultured for 24 h under hypoxic conditions. *P<0.05, when compared with NC.

Since the expression level of *miR-195* is increased under hypoxia, we transfected a *miR-195* inhibitor into hEPCs and the cells were harvested for qRT-PCR. The results indicate that the *miR-195* inhibitor could effectively suppress *miR-195* expression (Figure 2A). To determine the effect of *miR-195* on cell autophagy of hEPCs, the expression of the autophagy-related genes LC3B and beclin1 was examined using western blotting. The results showed that conversion of LC3B-I to LC3B-II and beclin1 expression was increased after *miR-195* inhibitor transfection compared with that of the NC transfected group under hypoxia (Figure 2B). Furthermore, to determine the effect of *miR-195* suppression on cell autophagy, the formation of autophagosomes was observed using TEM. As shown in Figure 2(C), the number of intracellular autophagosomes after *miR-195* inhibitor transfection was increased compared with that of the NC transfected group when cultured under hypoxic exposure. All these results showed that *miR-195* knockdown could promote the autophagy process of hEPCs under hypoxia.

### Inhibition of *miR-195* promotes cell proliferation, migration and angiogenesis of hEPCs under hypoxia

**Figure 3:**
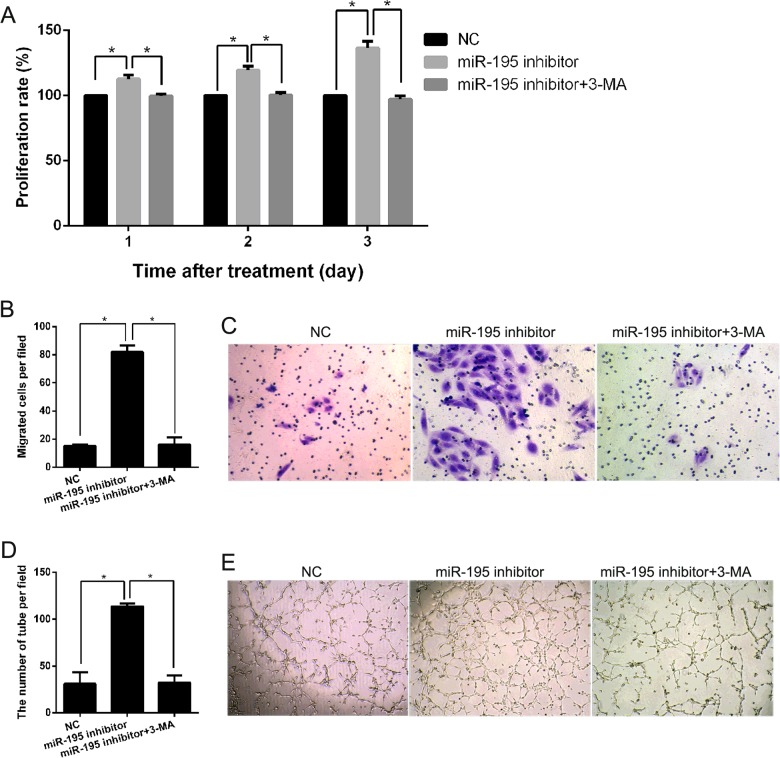
Inhibition of *miR-195* promotes cell proliferation, migration and angiogenesis of hEPCs through the autophagy process under hypoxia (**A**) The effect of the *miR-195* inhibitor or *miR-195* inhibitor plus 3-MA on cell proliferation under hypoxia as detected by the cell proliferation assay after transfection for 1, 2 and 3 days. (**B**) The average number of migrating cells per field among the indicated experimental groups is shown. (**C**) Representative images of EPC migration are shown. (**D**) The average number of tubes per field for the indicated experimental groups is shown. (**E**) Representative images of capillary tube formation are shown.

To investigate the effect of *miR-195* on cell proliferation, migration and angiogenesis of hEPCs under hypoxia, hEPCs were transfected with the *miR-195* inhibitor or NC under hypoxic conditions and cell proliferation, transwell migration and matrigel tube formation assays were carried out. As shown in Figure 3(A), transfection with the *miR-195* inhibitor could obviously promote the proliferation of hEPCs after transfection for 1, 2 and 3 days. For transwell migration assays, the number of cells that passed through the membrane into the lower chamber was significantly more for the *miR-195* inhibitor transfected cells than for NC transfected cells (Figures 3B and 3C). For the matrigel tube formation assay, hEPCs transfected with the *miR-195* inhibitor showed a significant improvement in capillary tube formation compared with that of the NC (Figures 3D and 3E).

To determine whether *miR-195* promotes cell proliferation, migration and angiogenesis of hEPCs through the autophagy process under hypoxia, hEPCs were treated with 3-MA which could block the autophagy process. The results showed that 3-MA treatment could counteract the effect of *miR-195* inhibitor on cell proliferation (Figure 3A), migration (Figures 3B and 3C) and capillary tube formation (Figures 3D and 3E).

### GABARAPL1 is a direct target of *miR-195*

**Figure 4:**
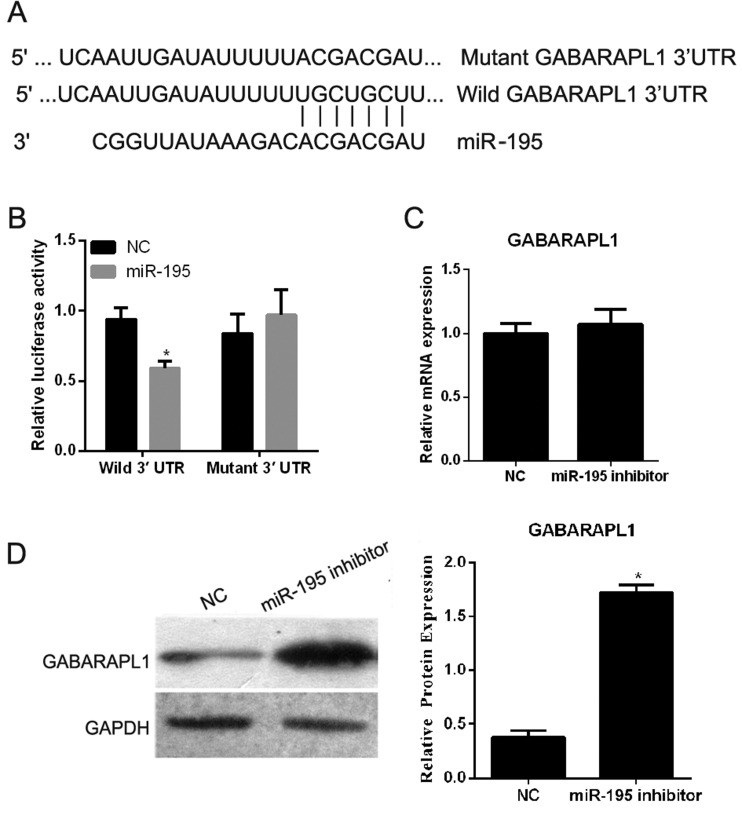
GABARAPL1 is a direct target of *miR-195* (**A**) Predicted duplex formation between the wild-type or mutant GABARAPL1 3′UTR and *miR-195*. (**B**) Luciferase activity of wild-type (3′UTR-Wild) or mutant (3′UTR-Mutant) GABARAPL1 3′UTR containing reporters, in 293T cells transfected with a *miR-195* mimic or NC. (**C**) qRT-PCR of GABARAPL1 mRNA in hEPCs transfected with a *miR-195* inhibitor or NC. Data were normalized to GAPDH mRNA. (**D**) Western blot of GABARAPL1 in hEPCs transfected with a *miR-195* inhibitor or NC. The GAPDH protein was used as an internal loading control. Left is representative images, and right is quantification of GABARAPL1 expression in each group presented in bar graphs as fold-increase. Data are expressed as the mean ± S.D., *P<0.05.

To elucidate the underlying mechanism by which *miR-195* regulates cell proliferation, migration and angiogenesis of hEPCs through autophagy, we explored *miR-195* targets using the microRNA.org bioinformatics algorithm. Our analysis revealed that GABARAPL1 was a potential target of *miR-195* based on putative conserved target sequences in the 178–184 bp region of the GABARAPL1 3′UTR (Figure 4A). To further examine whether *miR-195* directly targets GABARAPL1, a luciferase reporter assay was carried out and the results showed that a significant decrease in the luciferase activity of the reporter in the wild-type GABARAPL1 3′UTR containing vector was observed in the presence of *miR-195* (Figure 4B), compared with that of the NC. Such a significant decrease in reporter activity was not seen when the reporter was in the vector containing the mutant GABARAPL1 3′UTR (Figure 4B), despite the presence of *miR-195*, indicating that sequences in the 178–184 bp region of the GABARAPL1 3′UTR indeed interact with *miR-195*, inhibiting expression of GABARAPL1. We then examined the effect of *miR-195* inhibitor transfection on GABARAPL1 mRNA and protein levels. The inhibition of *miR-195* inhibition did not cause degradation of GABARAPL1 mRNA (Figure 4C). However, a clear increase in the level of endogenous GABARAPL1 protein was observed (Figure 4D).

### GABARAPL1 silencing decreased the effect of *miR-195* knockdown on the autophagy of hEPCs under hypoxia

**Figure 5:**
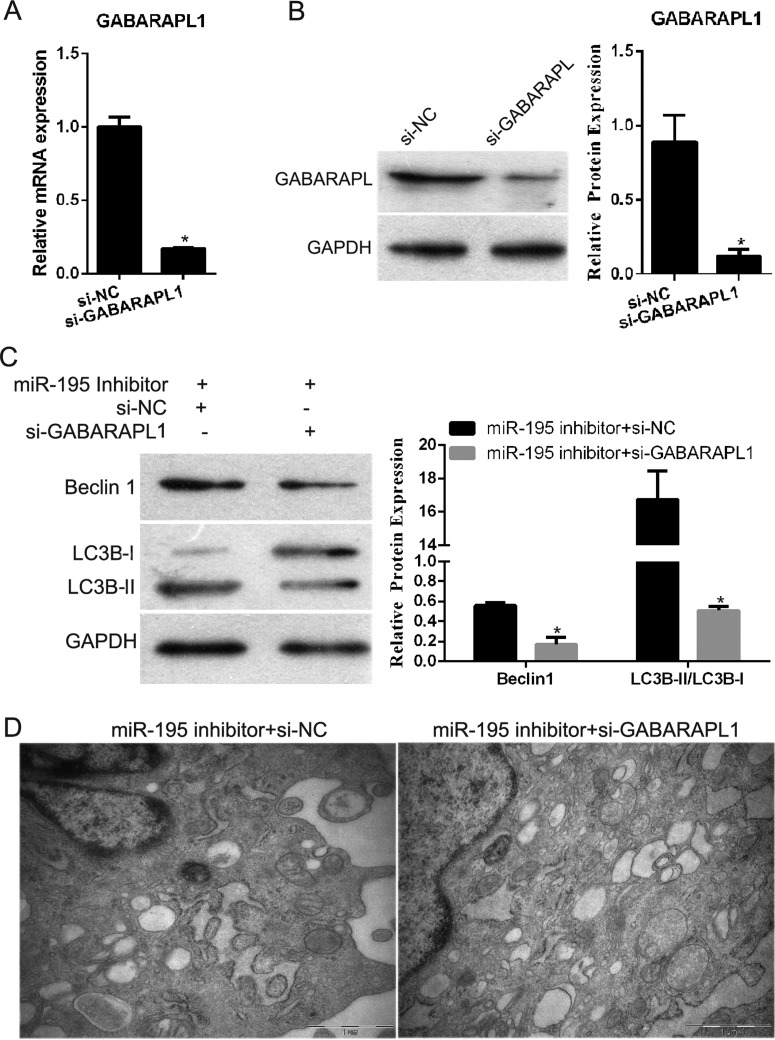
GABARAPL1 silencing decreased the effect of *miR-195* knockdown on the autophagy of hEPCs under hypoxia (**A**) The mRNA expression level of GABARAPL1 was successfully decreased after transfection with si-GABARAPL1. (**B**) The protein expression level of GABARAPL1 was successfully decreased after transfection with si-GABARAPL1. Left is representative images, and right is quantification of GABARAPL1 expression in each group presented in bar graphs as fold-increase. (**C**) The expression level of LC3B and beclin1 after transfection with si-GABARAPL1 plus *miR-195* inhibitor or si-NC plus *miR-195*. Left is representative images, and right is quantification of conversion of LC3B-I to LC3B-II (LC3B-II/LC3B-I) and beclin1 expression in each group presented in bar graphs as fold-increase. (**D**) The formation of autophagosomes after transfection with si-GABARAPL1 plus *miR-195* inhibitor or si-NC plus *miR-195* inhibitor as observed using TEM. *P<0.05.

To further investigate whether *miR-195* affected cell autophagy through its target GABARAPL1, GABARAPL1 was knocked-down using si-GABARAPL1 (Figures 5A and 5B). Then, the effect of si-GABARAPL1 plus *miR-195* inhibitor on the expression of the autophagy-related genes LC3B and beclin1 was examined using western blotting. The results showed that conversion of LC3B-I to LC3B-II and beclin1 expression were decreased after si-GABARAPL1 plus *miR-195* inhibitor transfection compared with that of the si-NC plus *miR-195* inhibitor transfected group under hypoxia (Figure 5C). In addition, the formation of autophagosomes was observed using TEM. As shown in Figure 5(D), the number of intracellular autophagosomes after si-GABARAPL1 plus *miR-195* inhibitor transfection decreased compared with that of the si-NC plus *miR-195* inhibitor transfected group cultured under hypoxic conditions. These results showed that GABARAPL1 silencing could decrease the effect of *miR-195* knockdown on the autophagy of hEPCs under hypoxia.

### GABARAPL1 silencing decreased the effect of *miR-195* knockdown on cell proliferation, migration and angiogenesis of hEPCs under hypoxia

**Figure 6:**
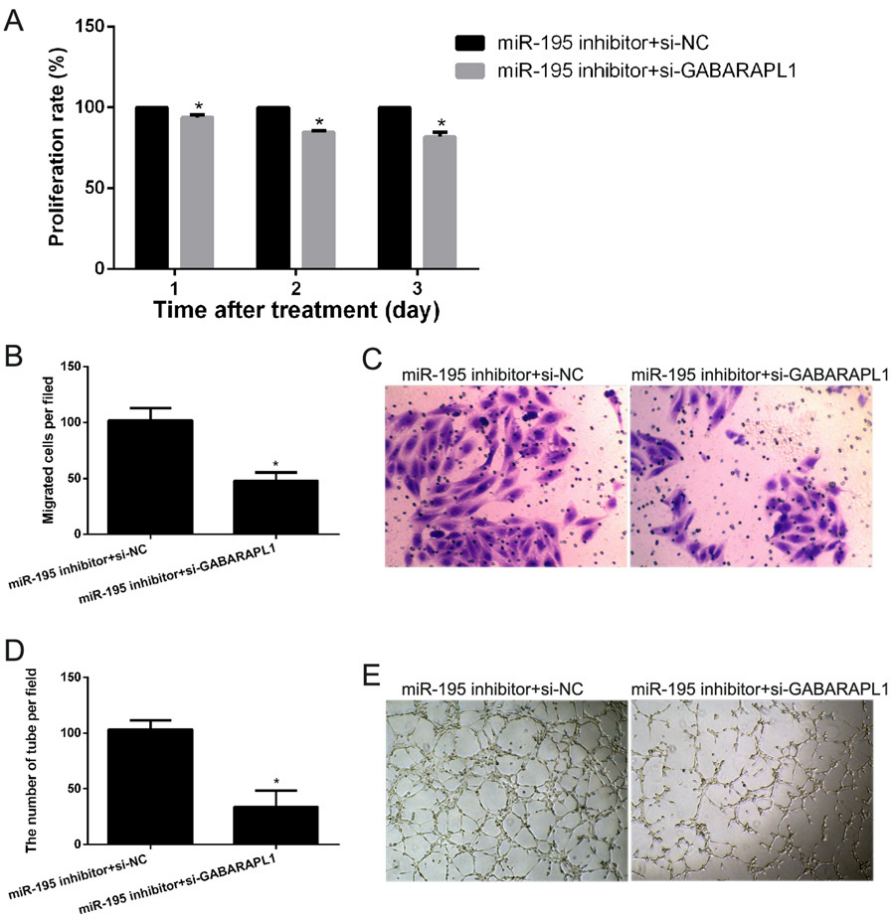
GABARAPL1 silencing decreased the effect of *miR-195* knockdown on cell proliferation, migration and angiogenesis of hEPCs under hypoxia (**A**) The effect of si-GABARAPL1 plus *miR-195* inhibitor or si-NC plus *miR-195* inhibitor on cell proliferation under hypoxia detected by cell proliferation assay after transfection for 1, 2 and 3 days. (**B**) The average number of migrating cells per field among for the indicated experimental groups is shown. (**C**) Representative images of EPC migration are shown. (**D**) The average number of tubes per field among the indicated experimental groups is shown. (**E**) Representative images of capillary tube formation are shown.

To investigate the effect of GABARAPL1 silencing on the effect of *miR-195* knockdown on cell proliferation, migration and angiogenesis of hEPCs under hypoxia, hEPCs were transfected with si-GABARAPL1 or si-NC plus *miR-195* inhibitor and cell proliferation, transwell migration and matrigel tube formation assays were carried out. As shown in Figure 6(A), the transfection of si-GABARAPL1 plus *miR-195* inhibitor could obviously suppress the proliferation of hEPCs after transfection for 1, 2 and 3 days compared with that of the si-NC plus *miR-195* inhibitor. For transwell migration assays, the number of cells that passed through the membrane into the lower chamber was significantly more for the si-GABARAPL1 plus *miR-195* inhibitor transfected cells than for the si-NC plus *miR-195* inhibitor transfected cells (Figures 6B and 6C). For the matrigel tube formation assay, hEPCs transfected with si-GABARAPL1 plus *miR-195* inhibitor showed a significant improvement in capillary tube formation compared with si-GABARAPL1 plus *miR-195* inhibitor (Figures 6D and 6E).

## DISCUSSION

In the present study, we firstly investigated the expression level of *miR-582*, *miR-195* and *miR-532* in hEPCs under hypoxic conditions. The highest increased expression and the known functions [16–21] of *miR-195* indicated that *miR-195* might play a role in regulating the function of hEPCs. Our subsequent assays verified this predication. The results showed that *miR-195* knockdown promotes the autophagy, cell proliferation, migration and angiogenesis of hEPCs under hypoxia. Based on these results, we predicated *miR-195* might be a target for improving the function of hEPCs, although this predication needs more studies for further confirmation. In addition, our results are supported by the findings of previous studies. Firstly, *miR-195* has been shown to have a significantly increased expression pattern between DVT and control subjects [14]. Secondly, *miR-195* has been shown to inhibit proliferation of certain cell types including thyroid, myoblast, cancer, mesenchymal stromal/stem and medullary thymic epithelial cells [16,18,22–25]. Thirdly, *miR-195* has also been shown to suppress the migration of cancer cells [24]. Finally, *miR-195* was found to play an anti-angiogenic role in endothelial cells, mesenchymal stromal/stem cells and cancer cells [16,18,26].

miRNAs exert their phenotypic effect by regulating the expression of target genes by either inducing mRNA degradation or inhibiting mRNA translation through imperfect base-pairing with the 3′UTR of target mRNAs [9,10]. In the present study, GABARAPL1 was identified as a direct target of *miR-195*, which regulates its expression by inhibiting mRNA translation through imperfect base-pairing with the 3′UTR in hEPCs. This conclusion is supported by several findings: (1) a complementary sequence of *miR-195* was identified in the 3′UTR of GABARAPL1 mRNA suggesting this 3′UTR interacts with *miR-195*; (2) inhibition of *miR-195* led to a significant reduction in GABARAPL1 protein expression; (3) over-expression of *miR-195* suppressed the luciferase reporter activity of the GABARAPL1 3′UTR- containing vector; (4) this effect was abolished by mutation of the *miR-195* binding site in the GABARAPL1 3′UTR and (5) silencing of GABARAPL1 decreased the suppressive effect of *miR-195* on autophagy, cell proliferation, migration and angiogenesis of hEPCs.

GABARAPL1 is a member of GABARAP family. The known function of GABARAPL1 was involved in regulating autophagy [27]. Autophagy levels are very low under physiological conditions, but are up-regulated in response to stress, such as hypoxia [28,29]. Previous studies have demonstrated that autophagy could regulate cell proliferation, migration and angiogenesis [30,31]. Based on our present study, it is sure that *miR-195* could regulate autophagy process under hypoxic conditions. In addition, *miR-195* also could regulate cell proliferation, migration and angiogenesis of hEPCs under hypoxic conditions. However, there is no study to demonstrate that GABARAPL1 directly regulated cell proliferation, migration and angiogenesis until now. So we predicted that *miR-195* might regulate cell proliferation, migration and angiogenesis through cell autophagy by targeting GABARAPL1. In the present study, we found that *miR-195* knockdown promotes the autophagy of hEPCs under hypoxia and inhibition of the autophagy process through 3-MA treatment could counteract the effect of *miR-195* inhibitor on cell proliferation, migration and capillary tube formation. In addition, GABARAPL1 decreased the suppressive effect of *miR-195* on cell proliferation, migration and angiogenesis of hEPCs. One previous study showed that transcription factor E2-2 inhibits the proliferation of EPCs by suppressing autophagy [32]. This result indicated that autophagy could promote the proliferation of EPCs and was consistent with the findings of our present study. All these results indicated that *miR-195* might regulate cell proliferation, migration and angiogenesis of hEPCs through autophay. However, this predication need more study to support.

In conclusion, hypoxia could induce the expression of *miR-195* and GABARAPL1 is a directed target of *miR-195*. The knockdown of *miR-195* promoted cell proliferation, migration, angiogenesis and autophagy of hEPCs under hypoxic conditions. Simply put, *miR-195* regulates proliferation, migration, angiogenesis and autophagy of hEPCs by targeting GABARAPL1.

## Abbreviations

DVT: deep vein thrombosis
EPC: endothelial progenitor cell
GABARAPL1: GABA type A receptor associated protein like 1
hEPC: human EPC
NC: negative control
qRT-PCR: quantitative real-time PCR analysis
